# The growth and pathogenesis of *Citrobacter rodentium* are compromised when multiple mucin sugar utilization pathways are disrupted, leading to accumulation of N-acetylglucosamine 6-phosphate

**DOI:** 10.1128/iai.00545-25

**Published:** 2026-03-16

**Authors:** Zhiquan Clarence Huang, Matthias Ahmad Mslati, Caixia Ma, Hyungjun Yang, Qiaochu Liang, Shauna Crowley, Ashley Gilliland, Roger Dyer, Irvin Ng, Hongbing Yu, Bruce A. Vallance

**Affiliations:** 1 Division of Gastroenterology, Hepatology and Nutrition, Department of Pediatrics, The University of British Columbia 195987, Vancouver, British Columbia, Canada; 2 Department of Microbiology, Molecular Genetics and Immunology, The University of Kansas Medical Center 21638, Kansas City, Kansas, USA; University of California San Diego School of Medicine, La Jolla, California, USA

**Keywords:** *Citrobacter rodentium*, attaching and effacing pathogens, mucin-derived sugars, amino sugar metabolism, N-acetylglucosamine, N-acetylneuraminic acid, sugar-phosphate stress, cell wall integrity

## Abstract

Many enteric bacterial pathogens, including attaching/effacing (A/E) *Escherichia coli* strains, cause acute gastroenteritis in humans. Considering the highly competitive nature of the mammalian gastrointestinal (GI) tract, these pathogens must rely on specific metabolic adaptations to establish successful infections. We hypothesized that A/E pathogens exploit host-derived nutrients within GI mucus, including N-acetylglucosamine (GlcNAc) and N-acetylneuraminic acid (NeuNAc), to fuel their pathogenesis. To address this hypothesis, we disrupted both GlcNAc and NeuNAc metabolic pathways in *Citrobacter rodentium,* a murine-specific A/E pathogen, by deleting *nagA*, which encodes the GlcNAc-6-phosphate (GlcNAc-6P) deacetylase that converts GlcNAc-6P into glucosamine-6-phosphate (GlcN-6P). Compared to wild-type (WT) *C. rodentium*, the Δ*nagA* mutant showed severely attenuated colonization and pathogenesis in C57BL/6J mice. Although Δ*nagA* cannot catabolize GlcNAc and NeuNAc, its *in vivo* defect could not be explained by nutrient deprivation alone. Instead, Δ*nagA* exhibited higher levels of cytosolic GlcNAc-6P, slower growth rate when cultured *in vitro*, altered regulation of GlcN-6P synthesis, and increased susceptibility to cell wall-targeting stressors. Supplementation with glucosamine (GlcN, which can be directly converted into GlcN-6P) partially restored the growth and resistance to cell wall stress of Δ*nagA* without reducing GlcNAc-6P accumulation, indicating that dysregulated GlcN-6P synthesis rather than GlcNAc-6P toxicity underlies its phenotype. Together, these data reveal a previously unrecognized metabolic vulnerability in *C. rodentium* where the disruption of the GlcNAc and NeuNAc metabolic pathways, by inactivating NagA, creates a sugar-phosphate imbalance that compromises cell wall integrity and pathogen fitness. Hence, targeting sugar-phosphate stress responses may provide a new therapeutic strategy against GI bacterial pathogens.

## INTRODUCTION

The mammalian gastrointestinal tract (GI) contains a large and diverse community of microorganisms that coexist with the host under homeostatic conditions. To maintain GI health, goblet cells, a specialized subset of intestinal epithelial cells (IECs), synthesize and apically secrete mucins, forming a dense and adherent mucus barrier that physically separates luminal microbes from the underlying IECs (1). This mucus barrier is primarily comprised of the gel-forming secreted glycoprotein mucin-2 (Muc2), which represents a large protein backbone with central proline, threonine, and serine-rich domains that undergo dense O-glycosylation in the goblet cell’s Golgi apparatus (2). These O-linked glycans consist of five different monosaccharides: *N*-acetylglucosamine (GlcNAc), *N*-acetylgalactosamine (GalNAc), *N*-acetylneuraminic acid (NeuNAc), galactose, and fucose (3). While mucus serves as a physical barrier that limits microbial access to host tissue, it also forms a nutrient-rich habitat overlying the impermeable barrier layer (4). This niche mucus layer is heavily colonized by an array of commensals, such as *Bacteroides thetaiotaomicron* and *Akkermansia muciniphila*, that express glycoside hydrolases to cleave mucin glycans and release free sugars that can support the proliferation of these mucolytic bacteria (5, 6). Notably, these liberated monosaccharides can also be exploited as nutrient sources by nearby enteric pathogens, such as *Salmonella enterica serovar* Typhimurium and *Clostridium difficile*, offering them competitive advantages over commensal microbes by supporting their expansion within the niche mucus layer (7).

Recent studies suggest that attaching and effacing (A/E) pathogens, including enterohemorrhagic *Escherichia coli* (EHEC) and enteropathogenic *E. coli* (EPEC), dwell within and ultimately cross the intestinal mucus layer to establish infection (8). A/E pathogens are major contributors to infantile diarrhea and mortality worldwide (8–10). To colonize their host’s GI tract, A/E pathogens must not only compete with the diverse resident microbiota but also with commensal *E. coli*, which occupy the same nutritional niche (11). Bertin et al. showed that the pathogenic EHEC strain EDL933 consumed all five Muc2-derived monosaccharides more efficiently than commensal *E. coli* when exposed to bovine small intestinal contents, demonstrating a competitive metabolic advantage *in vitro* (12). Even so, the precise mechanisms by which pathogens utilize mucin sugars to promote their growth and how such interactions impact infection dynamics *in vivo* are poorly understood.

Direct human studies of EPEC and EHEC are not feasible, and conventional laboratory mice are highly resistant to these clinically important pathogens. Therefore, *Citrobacter rodentium,* a natural A/E murine pathogen, is widely used to model EPEC and EHEC infections in humans (13–15). To establish infection, *C. rodentium* must cross the protective colonic mucus barrier to reach the intestinal epithelium (16, 17). As a member of the *Enterobacteriaceae* family, *C. rodentium* lacks the glycoside hydrolase enzymes used by some commensal microbes to degrade mucin-bound glycans, meaning they are unable to grow on intact mucins (18–20). Recent work by Liang et al. demonstrated that the monosaccharide NeuNAc can promote the growth of *C. rodentium,* as well as stimulate the secretion of two autotransporters, Pic and EspC, which enhance *C. rodentium*’s capacity to degrade mucus and adhere to underlying IECs, facilitating its transition from a luminal to a mucosa-associated niche. A *C. rodentium* mutant unable to import NeuNAc showed significantly impaired colonization and caused attenuated pathology in mice, highlighting the important role of NeuNAc utilization in pathogenesis (21, 22). However, NeuNAc is only one monosaccharide component of the Muc2 glycan, and the relative contribution of other abundant sugars, such as GlcNAc, remains poorly defined. Notably, both NeuNAc and GlcNAc are catabolized via pathways that converge onto a shared intermediate, GlcNAc-6-phosphate (GlcNAc-6P), suggesting that perturbations in one pathway may impact the other. This led us to investigate the role of both NeuNAc and GlcNAc in *C. rodentium* colonization, with a focus on how disruption of these metabolic routes may affect pathogen fitness *in vivo*.

In this study, we investigated the mechanisms by which *C. rodentium* exploits free GlcNAc and NeuNAc within the murine colon, and how disruption of its metabolic pathways may impair *C. rodentium*’s fitness and infectivity. To test the role of NeuNAc and GlcNAc catabolism in *C. rodentium*, a mutant strain (Δ*nagA*) was constructed by deleting the *nagA* gene encoding GlcNAc-6P deacetylase. Much like previous studies in *S*. Typhimurium and *Enterobacter hormaechei* that reported severe colonization defects upon deletion of *nagA* (23, 24), *C. rodentium* Δ*nagA* was severely impaired in infecting its murine hosts. However, rather than reflecting an inability to utilize GlcNAc and NeuNAc as nutrients, we determined that the impaired colonization reflected the accumulation of GlcNAc-6P. Our study shows that *nagA* deletion caused a sugar-phosphate stress that dysregulates the normal synthesis of the cell wall precursor, glucosamine-6-phosphate (GlcN-6P), and increases *C. rodentium’s* susceptibility to antimicrobial and osmotic stress. Supplementation with glucosamine (GlcN), bypassing the reaction catalyzed by NagA, partially restored Δ*nagA* growth and its resistance. These findings reveal a novel mechanism for reducing *C. rodentium* fitness by disrupting sugar catabolism through mutation of NagA, which leads to sugar-phosphate-based impairments and provides a potential strategy to limit A/E pathogen infection.

## RESULTS

### *C. rodentium* utilizes specific monosaccharides that are abundant in the colonic mucus

**Fig 1:**
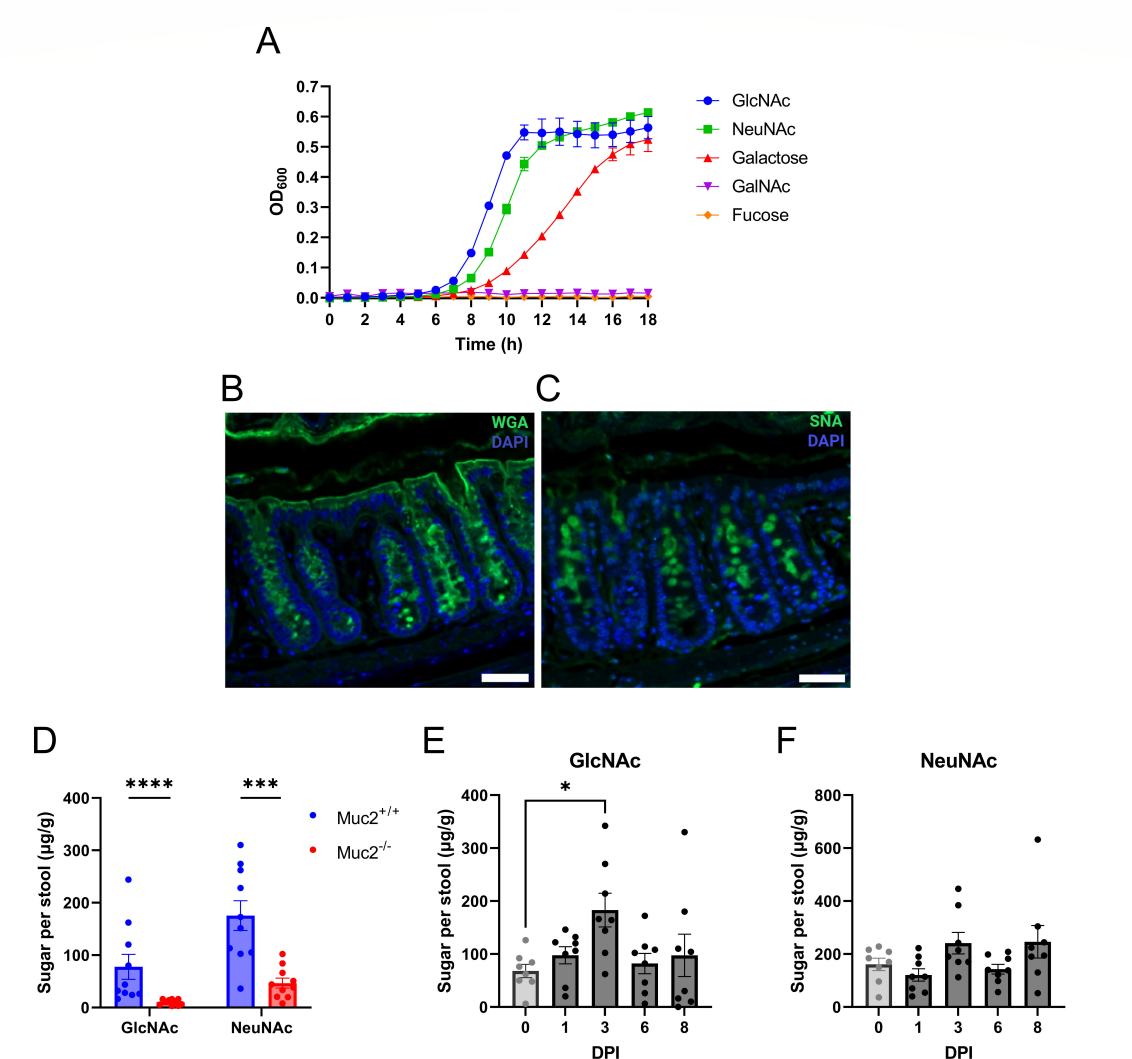
*C. rodentium* utilizes GlcNAc and NeuNAc that are enriched in the murine colon of C57BL/6J mice. (**A**) Growth assay of WT *C. rodentium* in minimal media with five monosaccharides constituting the Muc2 O-glycans. The absorbance at OD_600_ was measured every hour and was shown as mean ± SEM from biological triplicates. (**B**) Wheat germ agglutinin (WGA) and (**C**) Sambucus nigra agglutinin (SNA) staining of colonic cross sections from uninfected mice (at baseline). Sections were stained with WGA or SNA (green) to detect GlcNAc or NeuNAc, respectively, and 4′,6′-diamidino-2-phenylindole (DAPI) (blue) to detect DNA. Original magnification, 200×. Images are representative of three independent experiments with five mice per experiment (scale bar, 20 μm). (**D**) Levels of GlcNAc and NeuNAc in the stools of *Muc2^+/+^* and *Muc2^−/−^* littermates assessed by an ultra-high-performance liquid chromatography coupled with triple quadrupole mass spectrometry (UHPLC/QqQ-MS). Data were collected from 10 mice pooled from two independent experiments and shown as mean ± SEM. Statistical significance was determined by multiple Mann-Whitney tests. ****P < 0.0001, ***P < 0.001. Levels of (**E**) GlcNAc and (**F**) NeuNAc in the stool samples of mice at baseline and during infection with *C. rodentium* were assessed by UHPLC/QqQ-MS. Data were collected from eight mice pooled from three independent experiments and shown as mean ± SEM. Statistical significance was determined by Kruskal-Wallis tests (E and F). *P < 0.05. The Y-axis represents the amount of sugar in micrograms per gram of stool (**D–F**).

During its infection of the murine colon, *C. rodentium* initially colonizes the outer mucus layer, subsequently traversing the inner mucus barrier layer to infect underlying IECs, highlighting its close association with colonic mucus (16). Previous work by Liang et al. demonstrated that *C. rodentium* can grow on the mucin-derived sugar NeuNAc but not on intact mucin (22). Building on this work, we examined whether *C. rodentium* can utilize other mucin-derived sugars. We cultured wild-type (WT) *C. rodentium* in minimal media supplemented individually with each of the five major monosaccharides found in Muc2 O-glycans. As shown in Fig. 1A, *C*. *rodentium* grew robustly on GlcNAc and NeuNAc and, to a lesser extent, galactose but failed to grow on GalNAc or fucose. These results demonstrate that *C. rodentium* can utilize a subset of mucin-derived sugars for growth, particularly GlcNAc and NeuNAc, which appear to be preferred carbon sources.

We next tested whether GlcNAc and NeuNAc are present and accessible to *C. rodentium* within the murine colon. For these studies, we chose C57BL/6J mice, as they have been shown to be more susceptible to *C. rodentium* infection than other C57BL/6 substrains (25). To examine the spatial distribution of GlcNAc and NeuNAc, we stained colon tissue sections from C57BL/6J mice with the fluorescently labeled lectins, wheat germ agglutinin (WGA), which preferentially binds to GlcNAc, and Sambucus nigra agglutinin (SNA), which has an affinity for α-2,6-linked NeuNAc (26). WGA staining revealed a widespread signal within the cytoplasm of goblet cells, as well as strong labeling throughout the mucus layer lining the epithelial surface (Fig. 1B). In contrast, SNA staining appeared more punctate and enriched at the apical surface of goblet cells and in the outer mucus layer (Fig. 1C). While both lectins labeled goblet cells and the overlying mucus, their distinct patterns suggest differential localization of GlcNAc and NeuNAc within the mucus barrier, consistent with previous reports using WGA and SNA staining in the mouse colon tissues (22, 27).

To more precisely quantify GlcNAc and NeuNAc, we measured their levels within mouse fecal samples using an ultra-high-performance liquid chromatography coupled with triple quadrupole mass spectrometry (UHPLC/QqQ-MS). Because these sugars can originate from various sources, including exogenous food and host glycoproteins, we sought to determine whether they are primarily derived from secreted mucins. To do this, we compared wild-type (*Muc2^+/+^*) mice with *Muc2^−/−^* mice, which lack the major gel-forming mucin, Muc2, in the colon. Feces collected from *Muc2^+/+^* mice exhibited significantly higher levels of GlcNAc and NeuNAc as compared to *Muc2^−/−^* mice, supporting the conclusion that these sugars predominantly originate from Muc2 (Fig. 1D).

We next assessed whether *C. rodentium* infection altered the abundance of these sugars. Sugar quantification of fecal samples from infected C57BL/6J mice revealed that GlcNAc and NeuNAc remained detectable throughout the infection course (Fig. 1E and F), indicating their availability as potential nutrients. GlcNAc levels significantly increased by 3 days post-infection (DPI) compared to baseline, suggesting that *C. rodentium* infection may enhance the release of GlcNAc from mucin glycans. Together, these findings confirm that GlcNAc and NeuNAc are spatially enriched in the mucus layer, persist throughout infection, and are available for metabolic exploitation by *C. rodentium*.

### Inactivation of the GlcNAc and NeuNAc catabolic pathway impairs *C. rodentium* colonization in mice

**Fig 2:**
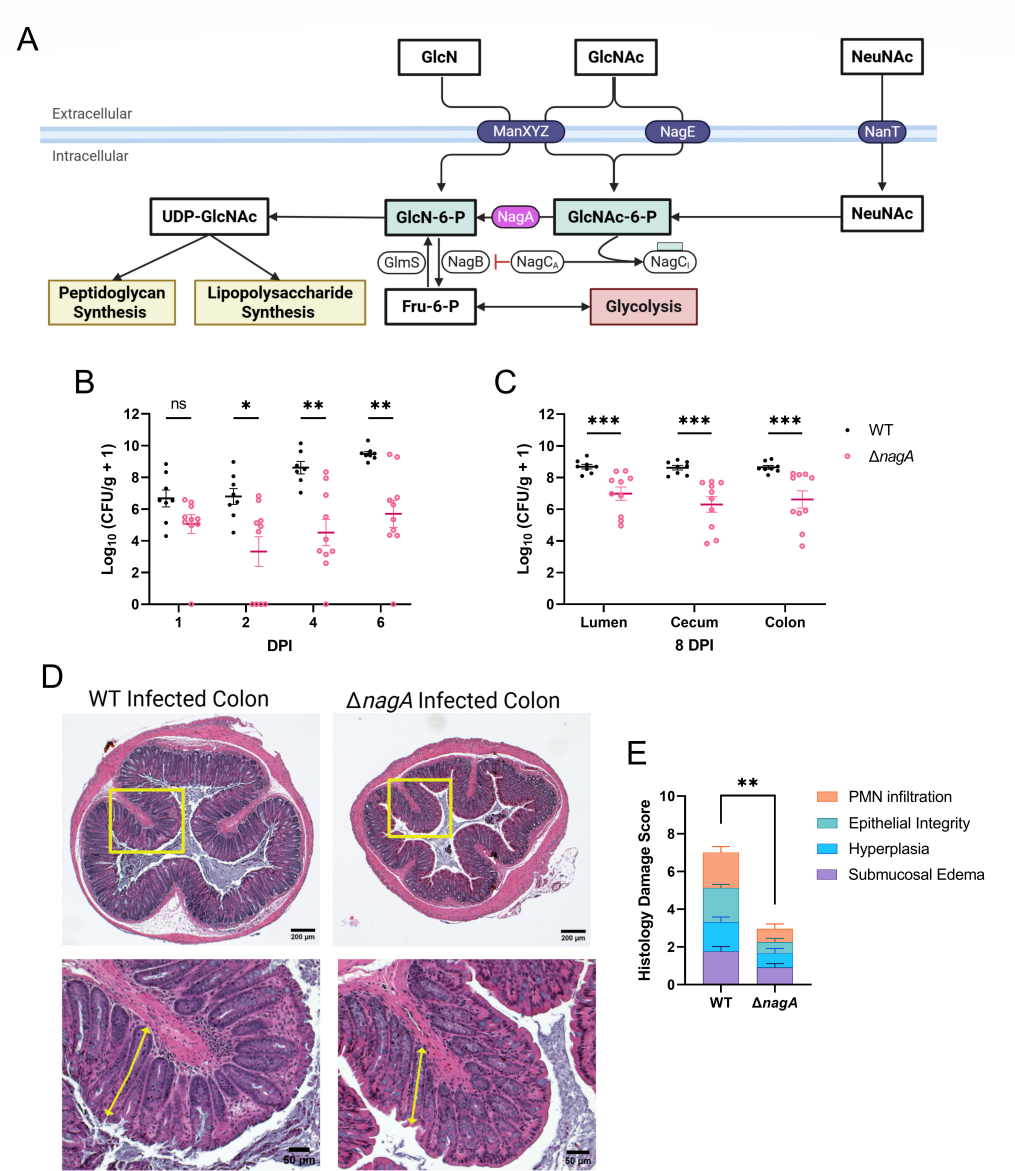
*C. rodentium* Δ*nagA* is significantly impaired in colonizing C57BL/6J mice intestine. (**A**) Schematic representation of the metabolic pathway of GlcNAc and NeuNAc in *C. rodentium*. NeuNAc is transported into the cell via the NanT transporter and converted into GlcNAc-6P, while GlcNAc is taken up by the ManXYZ and NagE PTS to directly form GlcNAc-6P. Inside the cell, GlcNAc-6P is deacetylated by NagA to generate GlcN-6P for glycolysis or peptidoglycan and lipopolysaccharide synthesis. (**B**) *C. rodentium* colony-forming unit (CFU) from stool pellets collected at 1, 2, 4, and 6 DPI and (**C**) luminal contents, as well as intestinal tissues, collected at 8 DPI from C57BL/6J mice orally infected with 10^8^ CFU of WT (*n* = 8) or Δ*nagA* (*n* = 10) *C. rodentium* (n, number of biological replicates). (**D**) Representative hematoxylin and eosin (H&E)-stained distal colon at 8 DPI for WT and Δ*nagA* mutant-infected mice (scale bar, 200 μm). Lower panels are expanded images of corresponding boxed regions in panels above. Crypt hyperplasia is indicated by arrows (scale bar, 50 μm). (**E**) Blinded histopathological scores of H&E tissue sections of mice infected with WT (*n* = 8) or Δ*nagA* (*n* = 10) *C. rodentium* (see Materials and Methods for scoring criteria). Agreement among raters was ensured by Spearman’s rank correlation coefficient r = 0.9283. Data were shown in mean ± SEM, and statistical significance was determined by multiple Mann-Whitney tests. ***P < 0.001, **P < 0.01, *P < 0.05 (**B, C, and E**).

The catabolic pathways for NeuNAc and GlcNAc are intricately linked among diverse microorganisms, with NeuNAc breakdown feeding directly into the GlcNAc pathway (28). Specifically, NeuNAc is first cleaved into N-acetylmannosamine and then converted into GlcNAc-6P, the same intermediate produced when extracellular GlcNAc is absorbed via the phosphotransferase system (PTS). In the bacterial cytoplasm, NagA further deacetylates GlcNAc-6P to form glucosamine-6-phosphate (GlcN-6P), a precursor for glycolysis and peptidoglycan synthesis (Fig. 2A).

To examine the role of GlcNAc and NeuNAc catabolism in *C. rodentium* colonization, we generated a mutant strain, Δ*nagA,* which lacks the NagA enzyme required to convert GlcNAc-6P into GlcN-6P. We infected C57BL/6J mice with either the WT or the Δ*nagA C. rodentium* strain and monitored pathogen shedding in fecal pellets at 1, 2, 4, and 6 DPI, followed by cecal and colonic tissue collection at 8 DPI. WT and Δ*nagA* infected mice exhibited similar low pathogen burdens at 1 and 2 DPI, harboring approximately 10^6^ colony-forming units (CFUs) per gram of stool (Fig. 2B). As the infection progressed, WT displayed a robust expansion to 10^9^ CFU by 6 DPI, while Δ*nagA* remained at levels 100–1,000-fold lower. This colonization difference persisted until 8 DPI, where WT *C. rodentium* exhibited significantly higher CFUs in the stool, as well as cecal and colonic tissues, as compared with the Δ*nagA* mutant strain (Fig. 2C).

Histological analysis revealed that the Δ*nagA*-infected mice developed a milder inflammatory response than WT-infected mice (Fig. 2D and E). While WT infection led to extensive epithelial damage, immune cell infiltration, and crypt hyperplasia, infection by the Δ*nagA* strain resulted in only limited epithelial cell disruption and reduced immune infiltration. These results demonstrate that the Δ*nagA* strain is impaired in its ability to colonize and induce pathological tissue damage in the colon.

### The attenuated phenotype of ΔnagA is driven by GlcNAc-6P accumulation rather than nutrient limitation

**Fig 3:**
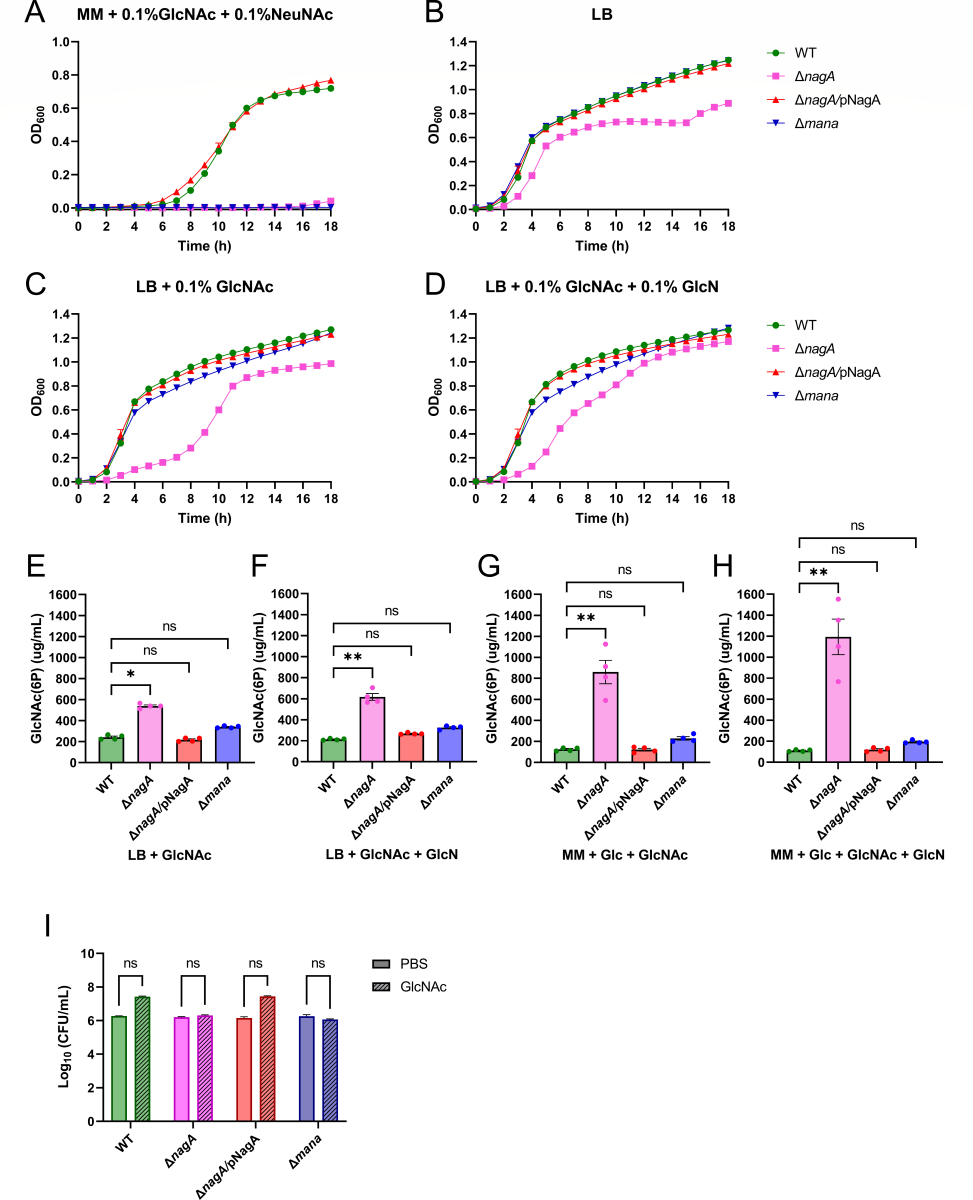
GlcNAc-6P accumulation leads to growth and colonization defects in Δ*nagA*. Growth assay of *C. rodentium* WT, Δ*nagA*, Δ*nagA/*pNagA, and Δ*mana* grown in (**A**) minimal medium supplemented with 0.1% GlcNAc and NeuNAc, and (**B**) LB media alone, or (**C**) supplemented with 0.1% GlcNAc and (**D**) 0.1% GlcN. The absorbance at OD_600_ was measured every hour, shown as mean ± SEM from biological triplicates. The levels of GlcNAc(6P) in the soluble extracts of the four *C. rodentium* strains in (**E**) LB with 0.1% GlcNAc and (**F**) GlcN or in minimal media (MM) with (**G**) GlcNAc and (**H**) GlcN were measured by the modified Morgan-Elson assay. Data were represented as mean ± SEM from biological quadruplicates. Statistical significance was determined by Kruskal-Wallis tests (**E–H**). **P < 0.01; *P < 0.05; ns, not significant. (**I**) Bacterial concentration of WT, Δ*nagA*, Δ*nagA/*pNagA, and Δ*mana C. rodentium* grown in PBS supplemented with or without 0.1% GlcNAc for 24h. Data were represented as mean ± SEM from biological triplicates. Statistical significance was determined by multiple Mann-Whitney tests.

As noted, previous studies have reported severe colonization defects of *S*. Typhimurium and *E. hormaechei* upon deletion of *nagA,* which were attributed to their inability to access GlcNAc and NeuNAc as nutrients (23, 24). To explore if this is the basis for the colonization defect observed with Δ*nagA C. rodentium*, we generated a mutant strain that cannot transport GlcNAc and NeuNAc into the cytoplasm. This mutant, lacking ManXYZ, NagE, and NanT transporters, was designated Δ*mana*. To rule out an unintentional polar effect created by the *nagA* deletion, we also generated a Δ*nagA/*pNagA complementation strain where the Δ*nagA* mutant was transformed with the plasmid pZA31MCS expressing NagA under its native promoter. WT, Δ*nagA,* Δ*nagA*/pNagA, and Δ*mana* were grown in minimal media supplemented with both GlcNAc and NeuNAc as carbon sources. WT and the Δ*nagA/*pNagA strain demonstrated similar growth rates, while Δ*mana* and Δ*nagA* failed to proliferate (Fig. 3A).

To examine whether the metabolic defect of Δ*nagA* could be rescued in nutrient-rich conditions containing abundant alternative carbon sources, such as glucose and amino sugars, we cultured all strains in LB media. Surprisingly, Δ*nagA* exhibited a distinct growth delay and failed to reach the same final optical density as the WT, Δ*mana*, or Δ*nagA/*pNagA strains (Fig. 3B). This growth delay was further exacerbated by supplementation with GlcNAc (Fig. 3C), but, interestingly, it was partially rescued by supplementation with GlcN (Fig. 3D). GlcN is a carbon source that can be transported by ManXYZ, a PTS transporter with broad specificity in sugars, including GlcNAc and GlcN, that feed into the GlcNAc/NeuNAc catabolic pathway and therefore bypass the need for NagA (29). Notably, Δ*nagA*, but not Δ*mana*, exhibited delayed growth in LB despite both strains being unable to catabolize GlcNAc and NeuNAc, indicating that impaired nutrient availability alone is insufficient to account for the growth defect. This phenotypic divergence between Δ*nagA* and Δ*mana,* which share the same inability to use GlcNAc and NeuNAc as carbon sources, suggested that differences in intracellular metabolic processing may underlie the Δ*nagA* phenotype.

We therefore examined the GlcNAc/NeuNAc metabolic pathway to identify distinguishing features between the two mutant strains. Δ*mana* cannot import GlcNAc or NeuNAc into the cytosol, whereas Δ*nagA* retains an intact transport system but cannot convert GlcNAc-6P to GlcN-6P, resulting in metabolic flux being halted at GlcNAc-6P (Fig. 2A). Based on these pathway differences, we hypothesized that Δ*nagA* may accumulate GlcNAc-6P intracellularly. To determine whether altered metabolite levels accompany the observed growth phenotypes, we quantified intracellular GlcNAc-6P levels using the Morgan-Elson assay under the same LB conditions used in the growth assays. This colorimetric assay detects N-acetylhexosamine, including both GlcNAc and GlcNAc-6P, via an alkaline borate reaction, followed by Ehrlich’s reagent to produce a chromogenic product measurable at 585 nm (30). Despite being unable to distinguish between free GlcNAc and GlcNAc-6P, the measured signals still primarily reflect intracellular GlcNAc-6P levels, as exogenously imported GlcNAc is always phosphorylated in the cytosol. When grown in LB supplemented with GlcNAc, Δ*nagA* accumulated significantly higher GlcNAc-6P than WT *C. rodentium*, while Δ*nagA*/pNagA and Δ*mana* showed levels comparable to WT (Fig. 3E). We next quantified GlcNAc-6P under minimal media conditions supplemented with glucose and GlcNAc as carbon sources, which provide a less variable metabolic background. Δ*nagA* again accumulated substantially higher levels of GlcNAc-6P compared to WT (Fig. 3G). Notably, supplementation with GlcN did not shift GlcNAc-6P levels in Δ*nagA* when grown in LB or minimal media conditions (Fig. 3F and H).

Following the observation that GlcNAc-6P accumulated in the Δ*nagA* mutant, we next assessed whether GlcNAc-6P accumulation produced a cytotoxic effect and reduced bacterial viability. WT, Δ*nagA*, Δ*mana*, and Δ*nagA/*pNagA *C. rodentium* strains were incubated in PBS supplemented with 0.1% GlcNAc for 18 h, and CFUs were enumerated before and after incubation. WT and Δ*nagA*/pNagA strains showed modest, but not statistically significant, increases in CFU over the incubation period, indicating no loss of viability under these conditions. More importantly, no significant decline in viability was observed in Δ*nagA,* similar to Δ*mana* (Fig. 3I), suggesting that GlcNAc-6P accumulation does not directly reduce the survival of Δ*nagA*.

Together, these data demonstrate that Δ*nagA* accumulates elevated levels of intracellular GlcNAc-6P under both nutrient-rich and minimal growth conditions, distinguishing it from Δ*mana* and the complemented strain. The persistence of GlcNAc-6P accumulation even under conditions that improve the growth of Δ*nagA* suggests there is an as-yet-undefined mechanism driving its phenotype.

### GlcNAc-6P accumulation disrupts normal GlcN-6P synthesis in ΔnagA, leading to compromised cell wall integrity

**Fig 4:**
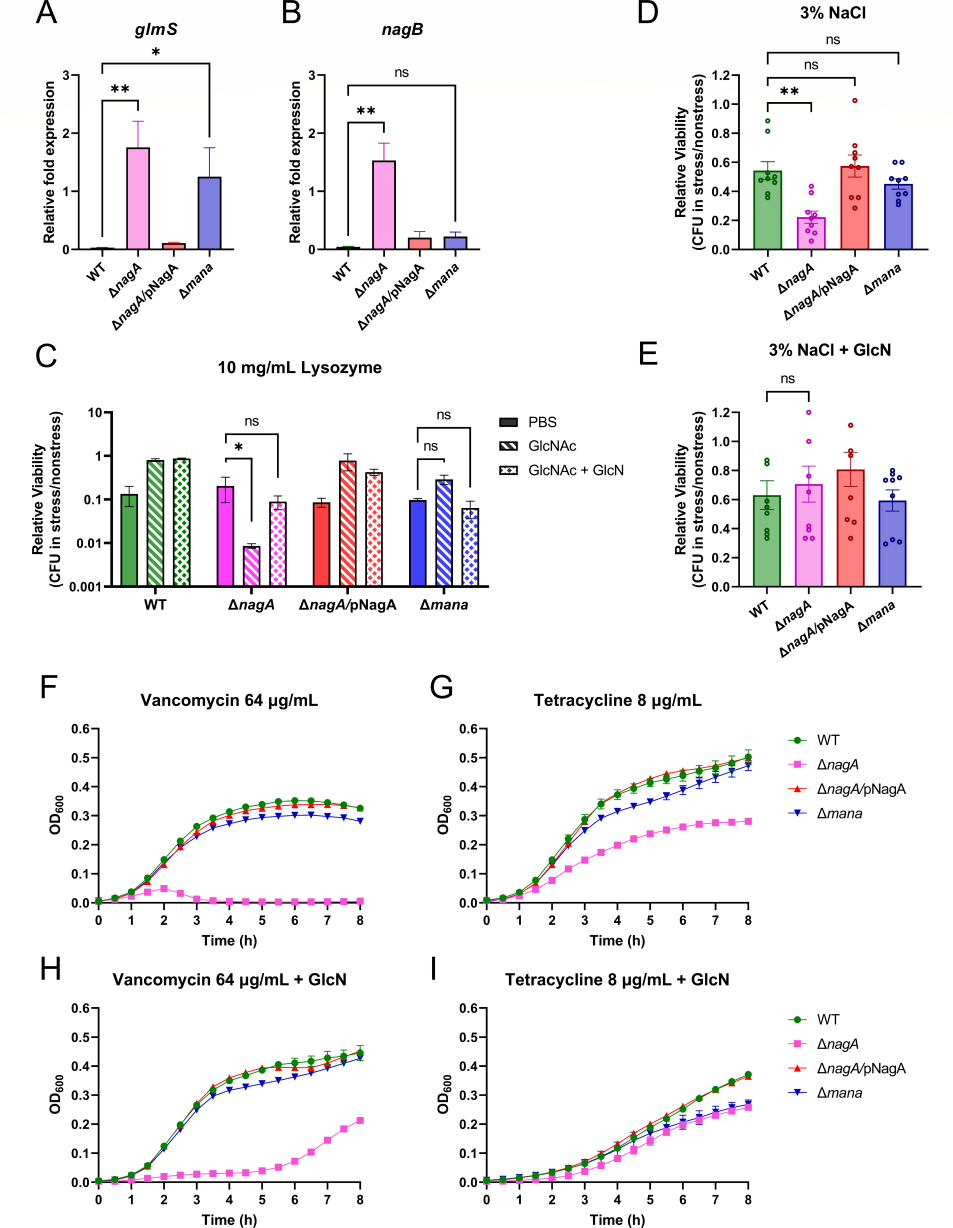
GlcNAc-6P accumulation depletes GlcN-6P, which leads to increasing sensitivity to cell envelope stressors. Bacterial qPCR analysis of genes responsible for (**A**) GlcN-6P synthesis from Fru-6P (*glmS*) and (**B**) conversion to Fru-6P (*nagB*). The expression level was normalized to the relative expression of the reference gene, *rpoA*, using the 2^−ΔCᴛ^. *n* = 4 (n, number of biological replicates). Data were collected from biological quadruplicates. (**C**) Survival of *C. rodentium* in the presence of 10 mg/mL lysozyme supplemented with/without 0.1% GlcNAc and GlcN. Relative viability was assessed by CFU quantification, normalized to PBS control groups. *n* = 3. Survival of *C. rodentium* in (**D**) LB or (**E**) LB with 0.1% GlcN under osmotic stress induced by 3% NaCl. Relative viability was assessed by CFU quantification, normalized to 1% NaCl control groups. *n* = 9. Statistical significance was determined by Kruskal-Wallis tests (**A–E**). **P < 0.01; *P < 0.05; ns, not significant. Growth curve of *C. rodentium* in LB with (**F and H**) 64 μg/mL vancomycin or (**G and I**) 8 μg/mL tetracycline supplemented with or without 0.1% GlcN. OD_600_ was monitored every half hour for a total of 8 h. *n* = 3. All the data were shown in mean ± SEM.

We next evaluated whether GlcNAc-6P accumulation in Δ*nagA* was associated with changes in expression of genes within the GlcNAc and NeuNAc catabolic pathway. It has been observed that Δ*nagA* growth was partially restored by the addition of GlcN in earlier assays (Fig. 3C and D). Since GlcN is transported by the ManXYZ phosphotransferase system, which mediates multiple sugar uptake, including GlcNAc and GlcN, we investigated genes responsible for GlcN-6P synthesis and turnover. Notably, while Δ*nagA* retains ManXYZ and can acquire exogenous GlcN-6P, the Δ*mana* strain lacks this transporter and relies solely on endogenous GlcN-6P production from fructose-6-phosphate (Fru-6P) via an aminotransferase GlmS, with its actions reversed by the deaminase NagB (Fig. 2A). Using RT-qPCR, we quantified transcript levels in *glmS* and *nagB* of *C. rodentium* grown in minimal media supplemented with GlcNAc. WT and Δ*nagA/*pNagA, which are able to generate GlcN-6P from exogenous GlcNAc, showed significantly lower *glmS* expression levels as compared with Δ*nagA* and Δ*mana*. In contrast, Δ*nagA* and Δ*mana* increased GlmS enzyme transcription levels to generate GlcN-6P from endogenous Fru-6P due to the incapability of taking up or catabolizing exogenous GlcNAc (Fig. 4A). However, it was observed that Δ*nagA* also significantly upregulates *nagB* compared with the other three strains (Fig. 4B). Previous studies in *E. coli* have shown that GlcNAc-6P accumulation deactivates the *nag* operon repressor, NagC, resulting in the derepression of *nagA* and *nagB* (31, 32). Consistent with the findings in *E. coli*, these results highlight a regulatory dilemma for *C. rodentium* Δ*nagA*: GlcNAc-6P accumulates but cannot be deacetylated to GlcN-6P, leaving *nagB* constitutively active to impede GlcN-6P synthesis. In contrast, Δ*mana* does not accumulate GlcNAc-6P and therefore avoids the transcriptional dysregulation observed in Δ*nagA* and can normally synthesize GlcN-6P from Fru-6P. Taken together, these results suggest that GlcNAc-6P accumulation in Δ*nagA* leads to the upregulation of *nagB* gene expression.

GlcN-6P is the essential substrate for peptidoglycan. We next assessed whether the compromised GlcN-6P synthesis could affect the integrity of the *C. rodentium* cell wall. Lysozyme is an innate antimicrobial enzyme found in the mammalian GI tract that hydrolyzes the β-1,4-glycosidic bond in the peptidoglycan backbone, thereby compromising bacterial cell wall integrity (33). To assess whether GlcNAc-6P accumulation sensitizes *C. rodentium* to lysozyme, we exposed our *C. rodentium* strains to 10 mg/mL lysozyme in the presence of GlcNAc or GlcN. It was observed that Δ*nagA* was more sensitive to the lysozyme challenge in the presence of GlcNAc, and such sensitivity was reduced when GlcN was added (Fig. 4C). This is consistent with our previous finding that GlcN supplementation allows Δ*nagA* to bypass NagA and enter the pathway as GlcN-6P, thereby alleviating the negative effect of GlcNAc-6P accumulation and restoring cell wall synthesis. Moreover, Δ*nagA* exhibited decreased viability under high NaCl concentrations (Fig. 4D), indicating that disrupted GlcN-6P synthesis can lead to high sensitivity to osmotic stress. Addition of GlcN restored this phenotype to WT levels (Fig. 4E), highlighting that direct replenishment of GlcN-6P bypasses the NagA-dependent step and rescues the Δ*nagA* defect.

Antibiotic susceptibility profiling further supported this cell wall defect. Δ*nagA* proved more vulnerable to vancomycin, a glycopeptide targeting the bacterial cell wall through blocking cross-linking by binding D-Ala-D-Ala termini of peptidoglycan precursors (Fig. 4F). Similarly, supplementation with GlcN resulted in a modest improvement in Δ*nagA* growth under vancomycin exposure (Fig. 4H). In contrast, Δ*nagA*’s susceptibility to tetracycline, a ribosome-targeting antibiotic that inhibits protein synthesis without directly affecting peptidoglycan, remained similar to the other three strains, with GlcN supplementation not significantly altering the strains’ responses in this assay (Fig. 4G and I). These results collectively demonstrate that GlcNAc-6P accumulation disrupts cell wall integrity through depleting GlcN-6P pools, providing a mechanistic explanation for the attenuated colonization observed in the *C. rodentium* Δ*nagA* strain. Altogether, GlcNAc-6P accumulation impairs GlcN-6P biosynthesis, disrupting peptidoglycan homeostasis and sensitizing Δ*nagA* to cell wall stressors.

### Deletion of GlcNAc/NeuNAc import rescues ΔnagA attenuation during mouse infection

**Fig 5:**
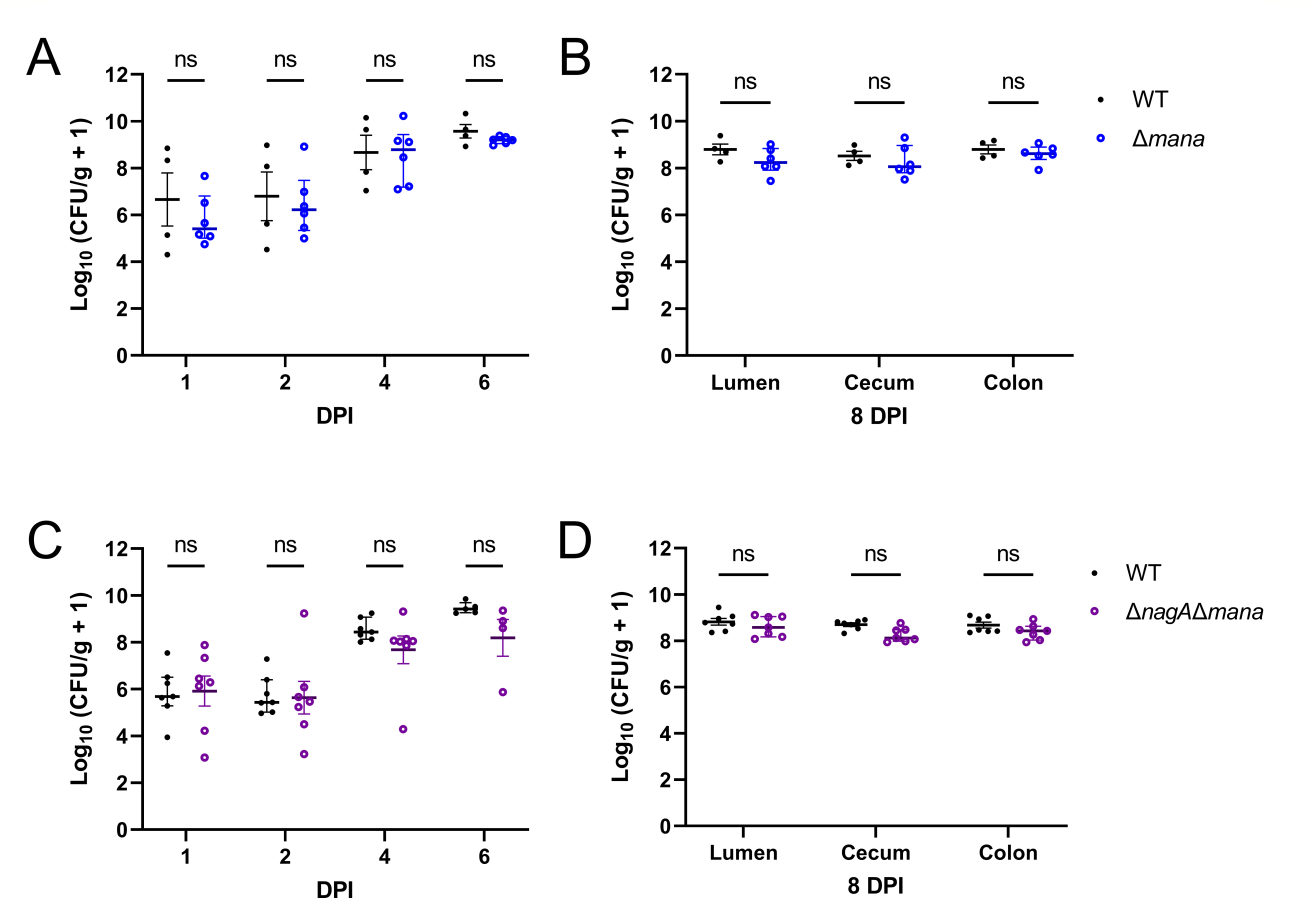
Preventing GlcNAc/NeuNAc import rescues Δ*nagA* attenuation during mouse infection. *C. rodentium* CFU from (**A**) stool pellets collected at 1, 2, 4, and 6 DPI and (**B**) luminal contents, as well as intestinal tissues, collected at 8 DPI from C57BL/6J mice orally infected with 10^8^ CFU of WT (*n* = 4) or Δ*mana* (*n* = 6) (*n*, number of biological replicates). *C. rodentium* CFU from (**C**) stool pellets collected at 1, 2, 4, and 6 DPI and (**D**) luminal contents, as well as intestinal tissues, collected at 8 DPI from C57BL/6J mice orally infected with 10^8^ CFU of WT (*n* = 7) or Δ*nagA*Δ*mana* (*n* = 7). Data were shown in mean ± SEM, and statistical significance was determined by multiple Mann-Whitney tests. ns, not significant.

The defect suffered by Δ*nagA C. rodentium* in colonizing the intestine of C57BL/6J mice could be due to the buildup of cytosolic GlcNAc-6P and/or insufficient usable nutrients (GlcNAc and NeuNAc). To distinguish between these two possibilities, we infected C57BL/6J mice with the Δ*mana* strain. The Δ*mana*-infected mice maintained high stool burdens and tissue bacterial loads comparable to WT (Fig. 5A and B), suggesting that the loss of GlcNAc/NeuNAc uptake alone does not significantly impair the ability of *C. rodentium* to infect mice under single-strain conditions. However, in a competitive infection assay, WT significantly outcompeted Δ*mana* (Fig. S1), demonstrating that access to GlcNAc/NeuNAc does provide a fitness advantage to *C. rodentium* during *in vivo* infection, but its impact is less than that seen with the Δ*nagA* strain.

We also assessed the colonization ability of the Δ*nagA*Δ*mana* mutant to determine whether loss of NagA impairs infection in the absence of GlcNAc/NeuNAc uptake. Similar to Δ*mana*, mice infected with Δ*nagA*Δ*mana* exhibited stool shedding and tissue burdens comparable to WT throughout the course of infection (Fig. 5C and D), suggesting that deletion of *nagA* alone is insufficient to reduce colonization when GlcNAc and NeuNAc cannot enter the cytosol. These findings thus demonstrate that the *in vivo* defect of the Δ*nagA* is due to the accumulation of GlcNAc-6P rather than their inability to uptake GlcNAc and NeuNAc, and blocking their uptake restores *C. rodentium* virulence despite the deletion of *nagA* during infection.

## DISCUSSION

**Fig 6:**
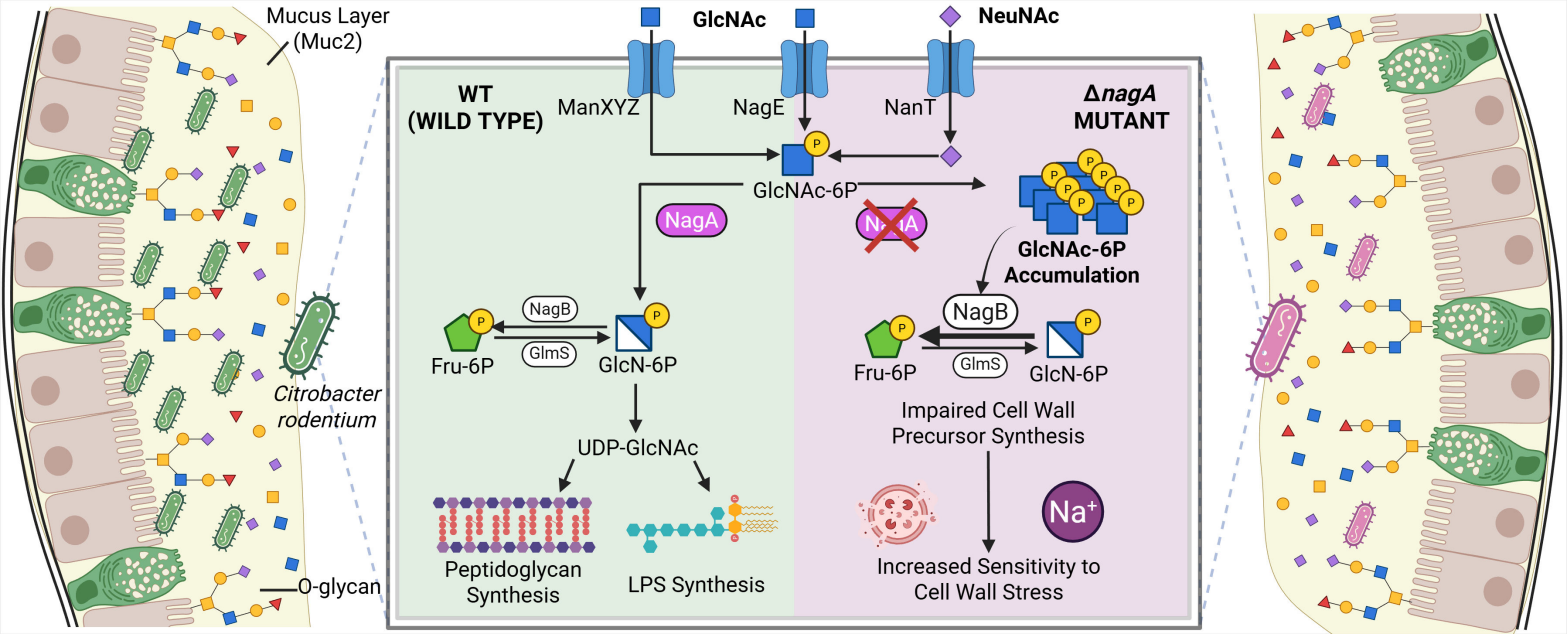
Proposed model illustrating how mucin-derived GlcNAc and NeuNAc support *C. rodentium* colonization and how *nagA* deletion disrupts this process. GlcNAc and NeuNAc released from Muc2 O-glycans are abundant within the colonic mucus layer and serve as readily accessible nutrients for *C. rodentium* during infection. In the WT strain (left), these sugars are transported into the cytosol via ManXYZ, NagE, and NanT and converted into GlcNAc-6P, which is further deacetylated by NagA to generate GlcN-6P. GlcN-6P feeds into UDP-GlcNAc biosynthesis and supports peptidoglycan synthesis, thereby maintaining cell wall homeostasis. In the Δ*nagA* mutant (right), GlcNAc-6P cannot be processed due to loss of NagA, leading to its intracellular accumulation and altered regulation in GlcN-6P synthesis. Reduced GlcN-6P availability limits cell wall precursor synthesis and increases susceptibility to lysozyme-, osmotic stress-, and vancomycin-induced damage, contributing to impaired colonization *in vivo*. The figure was generated on BioRender.com.

Our findings demonstrate that disruption of the sugar metabolism enzyme NagA impairs *C. rodentium*’s colonization and ability to cause colonic pathology in infected mice. Notably, several recent studies have identified similar major virulence defects in *S*. Typhimurium and *E. hormaechei* (23, 24). Strikingly, we determined that this defect in pathogenesis was not due to an inability to use the mucin-derived sugars GlcNAc and NeuNAc as nutrients, but instead reflects the creation of a metabolic bottleneck, driving the accumulation of GlcNAc-6P, which further disrupts the biosynthesis of GlcN-6P. With reduced cellular GlcN-6P levels, Δ*nagA C. rodentium* exhibited delayed cell growth and compromised cell wall integrity characterized by increasing sensitivity to lysozyme, osmotic pressure, as well as cell wall-targeting antimicrobials, ultimately resulting in decreased fitness and impairing its ability to establish infection *in vivo* (Fig. 6).

Intestinal mucus is primarily composed of the glycoprotein Muc2. Mucus can function as a physical barrier, preventing luminal microbes from accessing the underlying host tissues. Studies have shown that mice deficient in Muc2 or possessing a thinner-than-normal mucus layer are more susceptible to enteric infections (16, 34). *In vitro* experiments further demonstrate that mucus inhibits the attachment and translocation of the A/E pathogen EPEC (35–37). However, mucus can also serve as a habitat and provide nutrient sources for certain types of enteric bacteria (38). For example, the mucin-degrading commensal bacterium, *Akkermansia muciniphila*, can modulate the thickness of mucus by secreting mucolytic enzymes, which liberate mucin-derived sugars, including GlcNAc (39). These freed mucin sugars then become accessible carbon sources not only for beneficial commensal microbes but also for enteric pathogens, promoting their expansion and virulence, as shown in *S*. Typhimurium (7), *E. coli* (12, 19, 40, 41), *C. difficile* (7), and *Yersinia enterocolitica* (42).

Building on this broader understanding of mucin sugar utilization, our findings suggest that *C. rodentium* is well-adapted to this nutrient niche. Prior work has shown that *C. rodentium* preferentially colonizes colonic crypts enriched in Muc2-secreting goblet cells (43). Consistent with this, we found that *C. rodentium* can utilize three of the five Muc2-linked sugars as sole carbon sources for growth, with a pronounced preference for GlcNAc and NeuNAc over galactose (Fig. 1A). This selectivity might offer a competitive advantage to *C. rodentium* in a nutrient-limited intestinal environment, consistent with Freter’s hypothesis that pathogens must efficiently compete for limited substrates to avoid being flushed out by intestinal flow (44). This is especially relevant for *Enterobacteriaceae* pathogens, including *C. rodentium*, which must traverse or inhabit the mucus layer before infecting the underlying intestinal epithelium (16, 45). To assess the availability of GlcNAc and NeuNAc in the murine intestine, we used lectin staining to demonstrate the spatial distribution (Fig. 1B and C). While lectins bind glycan motifs rather than individual monosaccharides—e.g., WGA preferentially binds to terminal GlcNAc residues in dimer and trimer forms (46), and SNA recognizes NeuNAc linked to terminal galactose (47), they remain useful tools for mapping these sugars in colonic tissues. The primary source of free GlcNAc and NeuNAc in the lumen was confirmed to be the Muc2 mucin, as *Muc2^−/−^* mice displayed significantly lower levels of these sugars in their feces, as compared to *Muc2^+/+^* mice (Fig. 1D). Moreover, GlcNAc and NeuNAc remain available in the colon throughout *C. rodentium* infection and may serve as accessible nutrients that support *C. rodentium*’s growth and persistence *in vivo* (Fig. 1E and F).

The severe attenuation of the Δ*nagA* mutant during infection in C57BL/6J mice highlights the importance of functional GlcNAc/NeuNAc metabolism for pathogen fitness *in vivo* (Fig. 2B through E). However, because Δ*nagA* colonizes the intestine at such dramatically lower levels than WT, the limited pathological damage it causes may simply reflect its reduced pathogen burdens. Although deletion of *nagA* abolishes *C. rodentium*’s ability to catabolize GlcNAc and NeuNAc, our data demonstrate that it is not the nutrient deprivation *per se* but rather the buildup of metabolic intermediates, GlcNAc-6P, that disrupts the normal GlcN-6P synthesis in *C. rodentium* and thereby results in impaired fitness. Specifically, loss of NagA creates a metabolic chokepoint that results in the intracellular accumulation of GlcNAc-6P, which further impedes normal GlcN-6P synthesis and compromises cell wall integrity in *C. rodentium*. In the intestinal environment, where GlcNAc and NeuNAc are abundant and continuously released from Muc2 glycans, this metabolic bottleneck is likely exacerbated, promoting sustained GlcNAc-6P accumulation. These findings highlight a potential limitation in interpreting the importance of specific nutrients solely through catabolic gene deletions, as such mutations may also lead to unintended metabolic imbalances or chokepoints. For example, Sinha et al. highlighted the importance of GlcNAc for *E. hormaechei* infection as demonstrated by the reduced colonization of Δ*nagA* in neonatal mice (24). Similarly, Schubert et al. described GlcNAc as a context-dependent nutrient source based on the findings that Δ*nagB S*. Typhimurium is less competent than WT *S*. Typhimurium in specific mouse models (23). The conservation of the *nagA* gene across *Enterobacteriaceae* suggests that the potential consequences of sugar-phosphate accumulation, particularly GlcNAc-6P, can probably occur in an array of enteric pathogens, including *E. hormaechei* and *S*. Typhimurium (Fig. S2). Our findings highlight the necessity for researchers studying bacterial sugar metabolism to consider the negative impact of metabolite accumulation, which may significantly influence pathogen fitness and virulence, rather than attribute colonization defects solely to a failure to exploit these sugars as nutrient sources.

A growth defect resulting from the deletion of *nagA* has been previously reported in *E. coli* (48), *Bacillus subtilis* (49)*, Gluconacetobacter xylinus* (50), and *Streptomyces coelicolor* (51). Unlike common monosaccharides, such as glucose, fructose, and galactose, whose phosphate derivatives have been well described as exerting inhibitory effects on the growth of *E. coli* and *S*. Typhimurium (52)*,* the precise mechanism of how GlcNAc-6P exerts sugar-phosphate stress remains poorly understood in Enterobacter species. In many cases, there are two hypothesized causes of sugar-phosphate stress. One is that the accumulation of sugar phosphates can directly pose a cytotoxic effect, potentially by forming harmful byproducts (53). A recent study found that GlcNAc-6P can be transformed into a cytotoxic structural analog of ribose in *Streptomyces* by NagA and a previously uncovered enzyme, NagS (54), which is not present in *C. rodentium*. In contrast to *Streptomyces*, where deletion of *nagA* relieves toxicity by preventing the formation of downstream toxic metabolites (54), our data support a different mechanism in *C. rodentium*, in which the buildup of sugar phosphates depletes essential downstream intermediates and eventually impairs biosynthesis (55). GlcNAc-6P accumulation was not associated with a detectable loss of bacterial viability (Fig. 3I). However, Δ*nagA* cells showed significantly increased transcription of *nagB*, indicative of NagC inactivation and enhanced GlcN-6P catabolism (Fig. 4B). These findings support a model in which the *nagA* deletion not only blocks the breakdown of GlcNAc and NeuNAc into GlcN-6P but also rewires transcriptional regulation to suppress the synthesis of GlcN-6P from Fru-6P. In contrast, despite being similarly unable to generate UDP-GlcNAc from exogenous GlcNAc or NeuNAc, the Δ*mana* strain does not accumulate GlcNAc-6P and therefore avoids the transcriptional dysregulation observed in Δ*nagA*.

Although Δ*mana* alone sustained high colonization levels (Fig. 5A and B), its competitive disadvantage against WT in co-infection studies suggests that access to GlcNAc and NeuNAc provides a fitness advantage to *C. rodentium* during infection (Fig. S1). We selected C57BL/6J mice for this study because they display lower microbiota diversity and reduced colonization resistance against *C. rodentium* when compared to the C57BL/6NCrl used in our prior study with Δ*nanT* (25). The reduced microbial competition in C57BL/6J may obscure the contributions of GlcNAc and NeuNAc due to broader nutrient availability and redundancy in the gut. Supporting this notion, Fabich et al. demonstrated that deletion of GlcNAc and NeuNAc transport genes in EHEC EDL933 compromised its fitness only when tested in direct competition with their isogenic parent strains in streptomycin-treated mice (56). These findings underscore the importance of GlcNAc and NeuNAc under competitive conditions, where nutrient utilization becomes a critical determinant. The behavior of the Δ*nagA*Δ*mana* double mutant further supports the concept that access to GlcNAc and NeuNAc is critical to *C. rodentium* during infection. Δ*nagA* accumulates considerably more GlcNAc-6P when grown in minimal media than in LB (Fig. 3E and G). In the intestine, where GlcNAc and NeuNAc are abundant, Δ*nagA* may therefore experience metabolic conditions more similar to minimal media, favoring GlcNAc-6P accumulation and increasing stress sensitivity. In contrast, when uptake of these sugars is blocked in the Δ*nagA*Δ*mana* strain, the Δ*nagA*-associated attenuation is lost, suggesting that import of GlcNAc and NeuNAc is important during *C. rodentium* infection.

This study found that abnormal synthesis of GlcN-6P sensitizes *C. rodentium* to lysozyme, osmotic stress, and cell wall-targeting antimicrobials (Fig. 4C through I). These phenotypes underscore the critical role of GlcN-6P in maintaining cell wall integrity, likely through its involvement in peptidoglycan biosynthesis. A similar phenomenon has been observed in *Listeria monocytogenes*, where deletion of the *nagA* homolog (*lmo0956*) led to impaired cell wall synthesis, altered cell morphology, reduced teichoic acid content, and heightened sensitivity to mutanolysin, colistin, and ceftriaxone (57). In *E. coli*, NagA is also central to the dedicated peptidoglycan recycling pathway, which salvages GlcNAc released during murein turnover for *de novo* synthesis of murein and lipopolysaccharide (58). These findings highlight a conserved role for NagA across different bacteria in maintaining a balanced pool of precursors critical for cell wall homeostasis and also support a potential therapeutic concept. By supplying exogenous sugar to the pathogen while simultaneously inhibiting the appropriate enzyme in that sugar utilization pathway, like NagA, pathogens may be forced to accumulate metabolic intermediates that deplete other essential metabolites and compromise their survival. For instance, pyrimirhodomyrtone was identified as a natural product that impairs *Staphylococcus aureus* growth by binding the NagA active site (59). Additionally, methyl phosphonamidate analogs mimic the tetrahedral transition state of NagA’s enzymatic reaction and act as tight-binding inhibitors when incubated with NagA purified from *E. coli* (60). Despite being conceptually attractive, its application *in vivo* would require strategies to selectively target pathogens without disrupting commensals (61). Future studies should explore identifying pathogen-specific inhibitors, potentially through differences in enzyme structure or regulation, and evaluate their ability to attenuate colonization and virulence. Moreover, combining such an intervention with existing cell wall-targeting antibiotics may further enhance therapeutic efficacy.

In summary, our findings show that disruption of GlcNAc and NeuNAc metabolic pathways negatively impacts *C. rodentium* colonization fitness. Upon deletion of *nagA*, GlcNAc-6P accumulates and disrupts the normal synthesis of GlcN-6P, which impairs *C. rodentium* cell wall integrity and sensitizes the bacteria to cell-wall targeting stressors. These findings highlight the role of metabolic context in shaping host-pathogen interactions *in vivo*. Exploiting this vulnerability by targeting key enzymes, such as GlcNAc-6P deacetylase, could enable the development of novel therapeutics against A/E pathogens and other enteric microbes.

## MATERIALS AND METHODS

### Bacterial strains and routine growth conditions

All bacterial strains used in this study are listed in Table S1. Bacteria were routinely cultured in Luria Broth (LB) at 37°C aerobically with shaking at 200 rpm. LB supplemented with 1.5% agar was used for solid medium growth with aerobic incubation at 37°C for 16–18 h. When required, the growth medium was supplemented with streptomycin (Catalog #S4014, Sigma-Aldrich) at 100 µg/mL.

### *In vitro* growth assays

To assess the ability of *C. rodentium* to utilize and grow on Muc2-associated monosaccharides, M9 minimal media was used and generated by first preparing a 10× autoclaved base media (37.07 g of Na_2_HPO_4_, 30 g of KH_2_PO_4_, 5 g of NaCl, and 10 g of NH_4_Cl per a liter), then diluted in ddH_2_O to a final concentration of 2 mM MgSO_4_, 0.1 mM CaCl_2_, and 0.1% wt/vol of the sugar source. The final concentration of sugars was selected based on the reported concentration of these sugars in the purified porcine gastric mucin and to mimic the nutrient-limited conditions in the intestinal environment (62, 63). Sugar sources include GlcNAc, NeuNAc, GalNAc, galactose, and fucose. To evaluate the susceptibility of *C. rodentium* to antibiotics, LB was used as the growth medium supplemented with 64 µg/mL vancomycin or 8 µg/mL tetracycline, which is half of the minimal inhibitory concentration for WT *C. rodentium*. To monitor the growth of *C. rodentium,* 200 µL of the growth media was added to each well of the clear-bottom 96-well plate. Overnight cultures of *C. rodentium* grown in LB were washed with PBS and added to the media with the optical density measured at 600 nm (OD_600_) starting at 0.01. The cultures were incubated in the automated Varioskan LUX multimode plate reader (Thermo Fisher, USA) at 37°C with orbital shaking at 180 rpm. The growth was monitored by the spectrophotometer at OD_600_ for 18 h, and data were analyzed on Skanlt Software (version 6.1) RE for Microplate Readers RE (version 6.1.0.61).

### Measurements of Muc2 sugars using UHPLC/QqQ-MS

Quantification of GlcNAc and NeuNAc within mouse feces was adapted from Xu et al. (64), with slight modifications in the extraction, derivatization, and LC-MS conditions. Briefly, fecal samples (~20–30 mg) were diluted 20-fold (final concentration 20 µL/mg) with distilled water and homogenized overnight at 4°C. Supernatants were clarified by centrifugation (21,000 × *g*, 20 min) and subjected to 1-phenyl-3-methyl-5-pyrazolone (PMP) derivatization. Standard curves were prepared from a mixture of GlcNAc, galactose (Gal), and glucose (Glc), while NeuNAc standards were prepared separately and added after derivatization (0.39–50 µg/mL). For derivatization, 50 µL of sample or standard was mixed with 200 µL of 20%–22% ammonia and 200 µL of 0.2 M PMP in methanol, incubated at 70°C for 30 min. NeuNAc (not derivatized) was added afterward. Final mixtures of 20 µL derivatized sample and 60 µL non-derivatized sample were diluted 25-fold with dH₂O and 10% acetonitrile before injection. Quantitation of the sample sugars was done using an external standard method.

UHPLC separation was performed on a Waters Xevo TQS triple quadrupole mass spectrometer equipped with a Waters H-class Acquity UHPLC system and a Waters Acquity UHPLC BEH C18 Waters Acquity BEH C18 column (2.1 × 100 mm, 1.7 µm) at 35°C with a flow rate of 0.4 mL/min. The aqueous mobile phase A was 5 mM ammonium acetate adjusted to pH 8.3 with ammonium hydroxide; mobile phase B consisted of 95% acetonitrile and 5% ammonium acetate buffer (vol/vol, pH 8.3). The optimized gradient was as follows: 0.0–7.0 min, 12%–15% B; 7.1–8.5 min, 99% B; 8.6–10.0 min, 12% B. All four compounds were baseline resolved, with retention times of ~0.5 min (NeuNAc), ~5.0 min (Glc), ~5.5 min (Gal), and ~6.5 min (GlcNAc).

Detection was performed on the MS in MRM mode. Transitions were 511.2→175 (Glc/Gal), 552.2→175 (GlcNAc), and 310.3→274.2 (NeuNAc), with respective collision energies of 25, 30, and 5 eV. All analyses were performed using MassLynx and QuanLynx software.

### Construction of *C. rodentium* mutants and complementation strains

In-frame deletion mutants of *C. rodentium* strain DBS100 (streptomycin-resistant) were constructed using overlap extension PCR (65) and allelic exchange (66) strategies as previously described (67). Briefly, genomic regions (~1 kb) flanking the target gene were amplified using primers listed in Table S2 with overlapping sequences for fusion (P2 and P3) and engineered restriction sites (P1 and P4). The two fragments were fused and cloned into the suicide vector pRE112, which confers chloramphenicol resistance for selection. The resulting construct was transformed into *E. coli* MFD λpir by electroporation and conjugated into the WT *C. rodentium* strain. Mutants were selected based on sucrose resistance and loss of chloramphenicol resistance, indicative of successful double crossover events, and were confirmed by PCR and Sanger sequencing using gene-specific check primers (PF and PR).

Genetic complementation was performed as previously described (68). Specific primers (Table S2) were used to amplify the native promoter (P1-comp and P2-comp) and coding sequence of *nagA* (P3-comp and P4-comp). The construct was cloned into the XhoI/BamHI sites of pZA31MCS (Expressys, Ruelzheim, Germany) (69) to generate the plasmid pNagA containing wild-type *nagA*. Plasmid pNagA was electroporated into the *C. rodentium* Δ*nagA* strain to produce the *C. rodentium* Δ*nagA*/pNagA complemented strain.

### *In vivo* mice infection studies

C57BL/6J (6 to 8 weeks old) female mice were obtained from the Jackson Laboratory and maintained at the BC Children’s Hospital Research (BCCHR) Institute. Mice were kept in sterilized cages with filter tops, handled in tissue culture hoods, and provided with autoclaved food and water under specific pathogen-free conditions. Sentinel animals were routinely tested for common pathogens. For infection, mice were orally gavaged with 0.1 mL of overnight LB culture (~2.5 × 10^8^ CFU) of *C. rodentium*. To monitor colonization, fecal samples were collected in PBS at specified time points, weighed, homogenized, serially diluted, and plated on streptomycin-containing LB agar plates. The CFU was counted and divided by stool weight.

For tissue collection, mice were euthanized at 8 DPI, and their large intestines were collected and divided into ceca, proximal, and distal colons. For histological studies, a portion of the ceca and distal colons was obtained and fixed in either 10% formalin or Methacarn solution (methanol:chloroform:acetic acid, 6:3:1, vol/vol), where tissue sections were obtained with retained luminal content to preserve the mucus architecture. Next, the remainder of the ceca and distal colons was opened to retrieve the luminal content and thoroughly washed in PBS. Tissue and luminal contents were placed in Eppendorf tubes with 1 mL PBS and beads for bacterial enumeration, similar to the stool samples described above.

### *In vivo* competitive assays

The *C. rodentium* WT-AC strain and the competitive assay were conducted with modifications for *in vivo* infection following the protocol previously described by Gilliland et al. (67). In brief, the WT-AC *C. rodentium* expressing a tetracycline-inducible amCyan fluorescent protein was used to compete with Δ*mana*. The overnight culture of the two strains was normalized to the same OD_600_ and mixed in a 1:1 ratio. A volume of 100 µL of the mixture was orally gavaged to mice. Stool samples were collected in the following days, serially diluted, and plated on streptomycin-containing LB agar plates with 0.2 μg/mL anhydrotetracycline (Fisher, Catalog #AAJ66688MA) to induce the expression of amCyan in WT-AC. Plates were imaged using the iBright FL1500 Imaging System (Thermo-Fisher USA) for visualization of fluorescent WT-AC. The number of Δ*mana* (nonfluorescent) colonies was determined by subtracting the number of fluorescent colonies from the total colony count. The number of colonies corresponding to WT-AC versus Δ*mana C. rodentium* was transformed into a fraction of 1 to calculate the competitive index value.

### Histology and scoring

For routine histology to determine tissue morphology and pathophysiology changes, cecal and colonic tissues were fixed in 10% formalin, paraffin-embedded, and sectioned at 5 µm and then stained with hematoxylin and eosin (H&E). Histopathological scores were determined by two blinded observers independently as previously described (70). In brief, readouts included submucosal edema (0, no edema; 3, profound edema), epithelial hyperplasia (score based on the percent change in crypt height compared to that of the control crypts; 0, no change; 1, 1% to 50% change; 2, 51% to 100%; 3, >100%), polymorphonuclear cell infiltration (0, none; 3, severe), and epithelial integrity (0, no damage; 1, 10 epithelial cells shedding per lesion; 2, 11 to 20 epithelial cells shedding per lesion; 3, maximum damage to epithelial surface as noted by crypt destruction and epithelial ulceration). The maximum possible score was 12.

### Lectin staining

Paraffin-embedded colonic tissue sections (5 μm) were deparaffinized by heating at 60°C for 15 min, cleared with xylene, and rehydrated with 100%, 95%, and 70% ethanol, followed by dH_2_O. Dewaxed and dehydrated colonic tissue sections were blocked with Donkey Serum buffer at room temperature (RT) for 1 h. Fluorescently labeled lectins were diluted in antibody dilution buffer and used for staining: WGA (Catalog #FL-1021, Vector Laboratories, 1:500) for GlcNAc, and SNA (Catalog #FL-1301, Vector Laboratories, 1:200) for NeuNAc. For negative controls, lectins were pre-incubated with 100 mM of their target sugars for at least 30 min in the dark, then applied to unstained sections. All staining was carried out at RT for 2 h. Slides were washed once in PBS, twice in PBS + 0.1% Triton X-100, and twice in PBS. Sections were then mounted using ProLong Gold Antifade reagent (Molecular Probes/Invitrogen) that contains 4′,6′-diamidino-2-phenylindole for DNA staining. Sections were viewed at 350, 488, and 594 nm on a Zeiss AxioImager microscope. Images were obtained using a Zeiss AxioImager microscope equipped with an AxioCam HRm camera operating through AxioVision software (Version 4.4).

### Intracellular GlcNAc(6P) quantification

The level of GlcNAc(6P) was estimated by a modification of the Morgan-Elson procedure improved by Plumbridge (71). Briefly, bacteria were grown in 25 mL minimal media supplemented with 0.1% Glc and GlcNAc for 24 h. Each sample was normalized to OD_600_ = 0.5 (10^7^–10^8^ CFU/mL) and then harvested by centrifugation at 4,200 rpm, 4°C for 10 min. Pellets were washed with 1.5 mL H_2_O, resuspended in 250 µL H_2_O, and placed in a boiling water bath for 5 min for metabolite extraction. A volume of 50 µL bacterial extracts were mixed with 75 µL of 2 M potassium tetraborate tetrahydrate (Catalog #P5754, Sigma-Aldrich). The extracts were boiled for 3 min, cooled to room temperature, and then incubated with 625 µL Ehrlich’s reagent, prepared by dissolving 1 g of 4-(dimethylamino) benzaldehyde (Catalog #109762, Sigma-Aldrich) in 1.25 mL HCl and 100 mL glacial acetic acid, for 25 min at 37°C. The reacted purple extracts were centrifuged at 13,000 rpm at 4°C for 5 min to cease the reaction and remove any precipitate. The absorbance of the extracts was measured at OD_585_. GlcNAc standards (125–2,000 µg/mL) were used to generate a standard curve for calculation.

### Lysozyme killing assay

Overnight cultures of *C. rodentium* were diluted in PBS to an OD_600_ of 0.01 (~10^6^ CFU/mL). The bacterial culture was then treated with 10 mg/mL lysozyme (Catalog #L6876, Sigma-Aldrich), with or without supplementation of 0.1% GlcNAc and GlcN as indicated. Samples were incubated for 18 h at 37°C. After incubation, samples were serially diluted and plated on streptomycin-containing LB agar plates for CFU enumeration. Relative viability was calculated by N/N_L_, where N and N_L_ represent CFU counts in the presence or absence of lysozyme, respectively.

### Osmolarity viability assay

Overnight cultures of *C. rodentium* were diluted into 3 mL of LB broth with either 1% or 3% NaCl to an initial OD_600_ of 0.01. After 3 h of incubation at 37°C, bacterial suspensions were serially diluted and plated on LB agar to determine CFUs. Relative viability was calculated by N_3_/N_1_, where N_3_ and N_1_ represent CFU counts in the 3% and 1% NaCl conditions, respectively.

### RNA extraction and quantitative PCR

Overnight cultures of *C. rodentium* were diluted and incubated in 7 mL LB broth at 37°C, starting at OD_600_ = 0.01, and were harvested when OD_600_ = 0.7. Total RNA was isolated using the RNeasy Mini kit (Qiagen) according to the manufacturer’s protocol, and the RNA concentration was measured at the NanoDrop spectrophotometer (Thermo Fisher). Extracted RNA was reverse transcribed using 5X All-In-One RT MasterMix (Applied Biological Materials). For quantitative real-time PCR (qPCR), the cDNA was diluted 1:5 in RNase/DNase-free water. A total of 5 μL of the diluted cDNA was added to the PCR mix containing 10 μL of SsoFast EvaGreen Supermix (Bio-Rad) and primers listed in Table S3 (300 nM). qPCR was performed on a Bio-Rad CFX connect Real-time PCR detection system with the following cycling conditions: denaturation at 95°C for 30 s, followed by 3 s of denaturation at 95°C, 5 s of annealing at 60°C, 5 s of extension at 65°C for a total of 39 cycles. mRNA transcript expression was normalized to the relative expression of the reference gene, *rpoA*, using the 2^−(ΔCt)^.

### Phylogenetic and genomic analyses

A phylogenetic tree of *Enterobacteriaceae* bacteria containing *nagA* (K01204; EC 3.2.1.49) was generated using AnnoTree v1.2 at the genus level. Searches for *nagA* were performed with a minimum of 95% identity, 95% subject alignment, 95% query alignment, and an E value cutoff of 1 × 10⁻⁵. This resulted in the selection of the following genera: Yersinia, Vibrio, Shewanella, Serratia, Salmonella, Proteus, Pasteurella, Pantoea, Klebsiella, Hafnia, Haemophilus, Escherichia, Erwinia, Enterobacter, Cronobacter, Citrobacter A, Citrobacter, and Aeromonas. In R (v4.5.0), duplicate species entries were removed, and for each genus, the total number of genomes and the number and percentage of genomes containing *nagA* were calculated. Genera were categorized by total genome counts (0–10, 11–50, and >50). A heatmap of the percentage of genomes containing *nagA* was generated using the pheatmap package (v.1.0.13), with genome count categories displayed as row annotations and percentage values shown directly on the heatmap.

### Statistical analyses

Statistical analyses were performed using GraphPad Prism version 9.5.0 (GraphPad Software Inc.). Differences between the two groups were evaluated using the Mann-Whitney U test (non-parametric). For multiple comparisons, statistical analysis was performed using the Kruskal-Wallis test (non-parametric), followed by Dunn’s test as a post hoc test. Differences at *P* < 0.05 were considered significant, with asterisks denoting significance in figures. The specific statistical test applied for each data set is noted in the corresponding figure legend.

## Data Availability

All study data are included in the article and/or supplemental material.

## SUPPLEMENTAL MATERIAL

Supplemental material iai.00545-25-s0001.docx Table S1 to S3; Fig. S1 and S2.

ASM does not own the copyrights to Supplemental Material that may be linked to, or accessed through, an article. The authors have granted ASM a non-exclusive, world-wide license to publish the Supplemental Material files. Please contact the corresponding author directly for reuse.
