# Supplementary material for: The growth and pathogenesis of Citrobacter rodentium are compromised when multiple mucin sugar utilization pathways are disrupted, leading to accumulation of N-acetylglucosamine 6-phosphate
Source: Infect Immun. 2026 Mar 16;94(4):e00545-25. doi: 10.1128/iai.00545-25 (PMC13081724; doi:10.1128/iai.00545-25)

# **Supplementary Materials**

**Table S1. Bacterial strains used in this study, their genotype, phenotype, antibiotic resistance and the source.**

| **Bacterial strain and genotype** | **Phenotype** | **Antibiotic Resistance** | **Source** |
| --- | --- | --- | --- |
| *C. rodentium* DBS100 | Wild-type and parent strain of all mutants from this study | Streptomycin | (72) |
| *C. rodentium* Δ*nagA* | Unable to process GlcNAc and NeuNAc | Streptomycin | This study |
| *C. rodentium* Δ*nagA/*pNagA | Complement strain of Δ*nagA* | Streptomycin  Chloramphenicol | This study |
| *C. rodentium* Δ*manXYZ nagE nanT* (Δ*mana*) | Unable to uptake GlcNAc and NeuNAc | Streptomycin | This study |
| *C. rodentium* Δ*nagA manXYZ nagE nanT* (Δ*nagA*Δ*mana*) | Unable to uptake and process GlcNAc and NeuNAc | Streptomycin | This study |
| *C. rodentium* WT-AC | Tetracycline-inducible amCyan integrated into wild-type *C. rodentium* gene *xylE* | Streptomycin  Tetracycline | (67) |

**Table S2. Primers for mutant construction.**

| **Mutant** | **Primers sequences (5’-3') (Restriction sites are underlined)** |
| --- | --- |
| Δ*nagA* | P1: GACTAGGTACCGTTGTCTGAGACCAGGTTACGCTT (KpnI site)  P2: GCCATTGGCGACAACAATCGCATGGTCAT  P3: GATTGTTGTCGCCAATGGCCTTGACGAAGTGCTGAGGAT  P4: GACAGTGAGCTCCTGATAGCCCTGCTTAAGCAGGTTCA (SacI site)  PF: CACGATGGAGCTGAAAGTTAAGAC  PR: CTGGCTTTGTTCGGCAATCTGAAT  P1-comp: TGACCTCGAGATTTATTCCCCCTACGA (XhoI site)  P2-comp: ATTCTTCACCTCAGAAAGTCAA  P3-comp: ACTTTCTGAGGTGAAGAATAGGTCTGTAATGAA  P4-comp: AGCTGGATCCTTCTCTTACTCAGTGACGA (BamHI site) |
| Δ*manXYZ* | P1: ATAGCATGCTTCACAAAACGCGTCAGC (SphI site)  P2: AACAGTCTTACACTTGCTACCTCCTTTATTATCG  P3: GGAGGTAGCAAGTGTAAGACTGTTGTCCACAACC  P4: ATATCTAGACCACTGCCCCCAGTAAACAT (XbaI site)  PF: TTCACGAAATTCATCGC  PR: TGATCGTAGATGGCGTA |
| Δ*nagE* | P1: GACTAGGTACCCAGCACCAGAATCATCACCTCTT (KpnI site)  P2: GACATTCAGTAAATCTGGCTGACCGAATCGCAGCA  P3: GCCAGATTTACTGAATGTCGTGGTTTGCAGCAACATCGA  P4: GACTACCTCTAGAGCATACATCCACAGAATCACCT (XbaI site)  PF: GTTAGTGTAACCGATTACACC  PR: CCGTCGAATTTCATATCGGTAAC |

**Table S3. Primers for qPCR.**

| **Gene** | **Forward Primer** | **Reverse Primer** |
| --- | --- | --- |
| *nagB* | TGATACGGGTACGGGAG | TGCTCAATGGTAACGCG |
| *glmS* | TATGACGCAGGCGATAAAGG | GGTCCCAGCTCGCTTAAAT |
| *rpoA* | GCGCTCATCTTCTTCCGAAT | CGCGGTCGTGGTTATGTG |

Supplementary Figure 2. The taxonomic distribution of *nagA* within the family *Enterobacteriaceae*. Heatmap shows the distribution of *nagA* in selected genera within the family *Enterobacteriaceae*. The far-left column depicts the number of genomes analyzed for each genus. The intensity of blue color represents the percentage of genomes in this genus that contain *nagA*.

Supplementary Figure 1. Competition assay between WT-AC and Δ*mana C. rodentium* in mice stool. C57BL/6J mice were infected with 1:1 ratio of WT-AC and Δ*mana C. rodentium* overnight cultures. Bars represent the CFU ratio of WT and Δ*mana* in stool samples. Data and error bars represent Mean ± SD from 2 separate experiments with total of 6 mice.


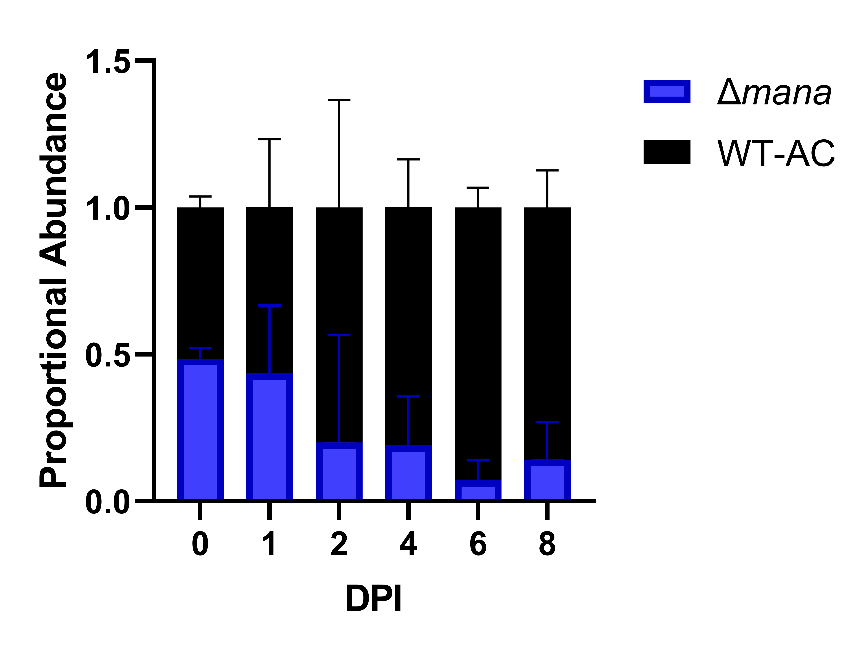

Supplement: Supplemental material — Table S1 to S3; Fig. S1 and S2. [file iai.00545-25-s0001.docx]
